# MRBENet: A Multiresolution Boundary Enhancement Network for Salient Object Detection

**DOI:** 10.1155/2022/7780756

**Published:** 2022-10-10

**Authors:** Xing-Zhao Jia, Chang-Lei DongYe, Yan-Jun Peng, Wen-Xiu Zhao, Tian-De Liu

**Affiliations:** College of Computer Science and Engineering, Shandong University of Science and Technology, Qingdao 266590, China

## Abstract

Salient Object Detection (SOD) simulates the human visual perception in locating the most attractive objects in the images. Existing methods based on convolutional neural networks have proven to be highly effective for SOD. However, in some cases, these methods cannot satisfy the need of both accurately detecting intact objects and maintaining their boundary details. In this paper, we present a Multiresolution Boundary Enhancement Network (MRBENet) that exploits edge features to optimize the location and boundary fineness of salient objects. We incorporate a deeper convolutional layer into the backbone network to extract high-level semantic features and indicate the location of salient objects. Edge features of different resolutions are extracted by a U-shaped network. We designed a Feature Fusion Module (FFM) to fuse edge features and salient features. Feature Aggregation Module (FAM) based on spatial attention performs multiscale convolutions to enhance salient features. The FFM and FAM allow the model to accurately locate salient objects and enhance boundary fineness. Extensive experiments on six benchmark datasets demonstrate that the proposed method is highly effective and improves the accuracy of salient object detection compared with state-of-the-art methods.

## 1. Introduction

The goal of salient object detection (SOD) is to find the most distinct and salient objects in an image. Salient object detection as an important preprocessing task in computer vision applications has been widely applied in many fields, such as semantic segmentation [1, 2], video segmentation [3], object recognition [4, 5], and cropping [6].

Inspired by cognitive studies of visual attention, most early works were based on handcrafted features, such as contrast [7, 8], center prior [9, 10], and so on [11–13]. With the improvement of GPU computing power, deep convolutional neural networks (CNNs) [14] have successfully broken the limits of traditional methods. These CNN-based SOD methods have achieved great success on widely used benchmarks.

**Figure 1:**
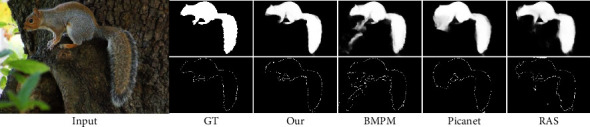
We compare our method with BMPM, Picanet, and RAS. The boundary maps in the figure are all calculated using the canny algorithm on their respective saliency feature maps.

Inspired by the excellent performance of FCN [15] based CNN in the field of semantic segmentation, FCNs have also been massively applied to SOD, such as several end-to-end deep network structures [16–18]. The basic units of salient object map finally output by these end-to-end network structures are the individual pixels in the image area, which can highlight the salient information. As the depth of the convolutional layer increases, the location of salient objects becomes more accurate. However, the detailed boundaries of salient objects are lost due to the pooling operation, see Figure 1.

Boundary information is critical for SOD. Therefore, many SOD jobs also try to enhance boundary details by different means. Some salient object detection models [19–21] refine high-level features with local information by combining U-Net with a bidirectional or recursive approach to obtain accurate boundary details. Some methods use preprocessing (superpixel) [22] and postprocessing (CRF) [17, 21] to preserve object boundary information. Besides, loss functions have also been used to obtain high-quality salient objects. For example, BASNet [23] uses the proposed hybrid loss to improve boundary accuracy. Some methods [24–26] attempt to use the edge as the supervision for training SOD models, which significantly improves the accuracy of the saliency map.

We explicitly model edge features and use the attention mechanism to fuse edge features and salient features to obtain salient objects with a high-quality edge.

Our contributions can be summarized as follows:

- We propose an MRBENet network that utilizes FFM to fuse salient edge features to enhance the boundary and semantic features of salient objects. From the top layer to the bottom layer, the edge details of salient features are sequentially optimized. When extracting edge features, the guidance of high-level semantic features can effectively avoid the influence of shallow noise. Experimental results show that it can filter out noise.
- The edge features are first supervised by the salient edge ground truth, and then fused with the salient object features through a feature fusion module. The feature aggregation module extends the receptive field through multi-scale convolution, which can not only effectively aggregate features but also promote feature fusion and enhance the edge details of salient features.

## 2. Related Work

Traditional nondeep learning methods predict salient objects based mainly on low-level features, such as pixel contrast [12], average image color difference [27], and phase spectrum with Fourier transform [28].

Compared with traditional methods, the convolutional neural network (CNN) performs extraordinarily. In [15], Long et al. first proposed a fully convolutional neural network (FCN) to predict each pixel. The FCN replaces the last fully connected layer of the convolutional network with a convolutional layer. At the end of the network structure, the feature map is up-sampled, and then the up-sampled feature map is classified into pixels. The final output is an end-to-end image.

In recent years, most neural network models for salient object detection have extended or improved fully convolutional neural networks. HED [29] added a series of side output layers after the last convolutional layer of each stage of VGGNet [30] and fuses the feature maps output by each layer to obtain the final result map. In DSS [17], Hou et al. added several short connections from the deeper side output to the shallower side output, so that higher-level features can help locate lower-level features, and lower-level features can enrich the details of higher-level features. The smart combination of higher and lower-level features makes it possible to detect salient objects accurately. PoolNet [26] made full use of the function of the pool and incorporated three residual blocks. Wu et al. [31] embedded a mutual learning module and an edge module in the model. Each module is separately supervised by the salient object and the edge of the salient object and is trained in an intertwined bottom-up and top-down manner. Wang et al. [32] designed a pyramid attention module for salient object detection and proposed an edge detection module. The former extends the receptive field and provides multi-scale clues. The latter uses explicit edge information to locate and enhance the saliency of the object edge. Wang et al. [33] proposed an iterative collaborative top-down and bottom-up reasoning network for salient object detection. The two processes of top-down and bottom-up are alternately executed to complement and enhance the fine-grained saliency and high-level saliency estimation. Noori et al. [34] proposed a multiscale attention guidance module and an attention based multilevel integrator module. These two modules not only extract multi-scale features but also assign different weights to multi-level feature maps.

Given the huge body of work in this field, the latest research progress of SOD can be quickly grasped through relevant surveys. In [35], Borji et al. comprehensively reviewed the works and development trends of salient object detection before 2019 and discussed the impact of evaluation indicators and dataset bias on model performance. Recently, Wang et al. conducted a comprehensive survey [36] covering all aspects of SOD. They summarized the existing SOD evaluation datasets and evaluation indicators, constructed a new SOD dataset with rich attribute annotations and analyzed the robustness and portability of the deep SOD model for the first time in this field.

Recently, RGB-D/RGB-T SOD is a growing trend. The accuracy of saliency detection can be improved by learning simultaneous multimodal information. For example, Ji et al. [37] proposed a depth calibration framework (DCF) learning strategy. DCF generates depth images quality weights by classifying positive and negative samples of depth images. The depth images are then calibrated based on these weights. Through this strategy, the accuracy of saliency estimation is improved by depth information, and it tackles the influence of bad information in depth images on saliency results.

## 3. Proposed Model

### 3.1. Features Extraction

**Figure 2:**
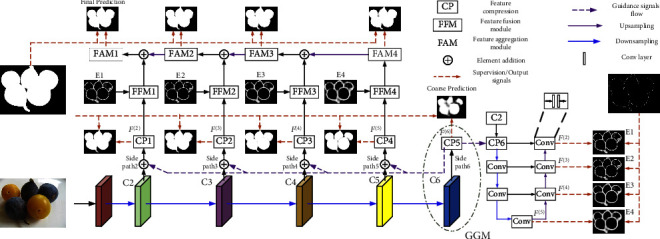
Framework of MRBENet. GGM (global guidance module) consists of a set of deeper and successive convolution layers and a CP module. The CP module consists of two convolution layers and a ReLU layer.

We use the vgg16 network as the backbone network. As shown in Figure 2, we delete the full connection layer in vgg16 and add a set of deeper successive convolution layers. The deeper convolution block and CP module form a GGM module for extracting and enhancing high-level semantic features. The high-level features can help locate the location of salient objects and edges. From the backbone network, we obtain six side features, Conv1-2, Conv2-2, Conv3-3, Conv4-3, Conv5-3, and Conv6-3, from the backbone network. The six side features can be denoted by the backbone feature set *C*:

(1) $$\begin{array}{c} C=\left\{C^{\left(1\right)},C^{\left(2\right)},C^{\left(3\right)},C^{\left(4\right)},C^{\left(5\right)},C^{\left(6\right)}\right\}\text{.} \end{array}$$

Side path1 is abandoned because side path1 is too close to the input image, so the receptive field is very small. In addition, encoding shallow features will significantly increase the computational cost [38]. Side path1 has little effect on the final result.

Since the side output features have different resolutions and number of channels, we first use a set of CP modules to compress the number of side output feature channels into an identical, smaller number, denoted as *k*. This is beneficial for reducing the amount of subsequent computation and for performing subsequent elementwise operations. The compressed side features can be expressed as follows:

(2) $$\begin{array}{c} F^{\left(i\right)}=f\left(\phi \left(Trans\left(C^{\left(i\right)};\theta \right)\right)\right)\text{,} \end{array}$$

where Trans(*C*^(*i*)^; *θ*) represents the convolutional layers with parameter *θ* (it can change the number of channels of the feature map), and *ϕ*(·) represents the ReLU activation function, *i* ∈ {2,3,4,5,6}.

Low-level features have rich information, but some of the information will interfere with the SOD task. So, it is necessary to highlight the salient information of low-level features. The added GGM has the largest receptive field. Therefore, we predict a coarse saliency map for this layer to guide the network to extract useful details from low-level features. The coarse prediction map can roughly locate the salient object regions having larger saliency values (weights) than the background regions. We upsample the coarse saliency map to make its resolution consistent with that of the low-level feature layers. In order to find the right details of salient objects in low-level features, we combine low-level features with coarse prediction maps to enhance the useful details of salient objects. The data flow indicated by the purple dotted arrow in Figure 2 represents the guidance of the coarse saliency map to the low-level features. *F*^(*i*)^ can be expressed as follows:

(3) $$\begin{array}{c} F^{\left(i\right)}=f\left(\phi \left(Trans\left(\left(C^{\left(i\right)}+Up\left(F^{\left(6\right)};C^{\left(i\right)}\right)\right);\theta \right)\right)\right)\text{,} \end{array}$$

where Up denotes the bilinear interpolation operation, *i* ∈ {2,3,4,5}.

We explicitly model the edge features on side path2. We utilize a U-shaped network to extract edge features at four different resolutions. This network consists of a CP module and six convolution blocks. *C*^(2)^ and *F*^(6)^ are elementwise summed and input into the network. The CP module compresses the input features into *k* channels. The convolution block consists of two convolution layers to enhance and extract edge features. We also add four edge supervisions to this network. As shown in Figure 2, we get the edge feature *F*_*e*_^(*i*)^, *i* ∈ {2,3,4,5}.

### 3.2. Features Fusion Module (FFM)

**Figure 3:**
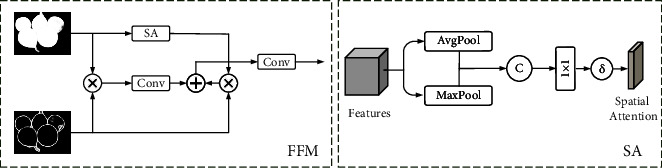
FFM: features fusion module. SA: spatial attention.

As shown in Figure 3, the Spatial Attention (SA) module performs maximum pooling and average pooling on features. Two pooling operations are used to aggregate the channel information of the features, and two single channel maps are obtained. The two images are concatenated together to get a spatial attention through a standard convolutional layer. Spatial attention focuses on the weights of each part of the feature map. Therefore, the spatial attention model can find the most important part (salient object) in the feature map, which is very suitable for SOD tasks.

The corresponding salient feature *F*_*S*_ and edge feature *F*_*E*_ are input into the FFM module for feature fusion. We first enhance the edge features in the salient features by multiplication. Then we use a 3 × 3 convolution layer to drive the preliminarily fused feature *P*, which can be expressed as follows:

(4) $$\begin{array}{c} P=conv_{3\times 3}\left(F_{S}\times F_{E}\right)\text{.} \end{array}$$

Meanwhile, we apply the spatial attention module to salient features to get a feature vector, then multiply it with the edge feature to obtain the feature ϒ, which can be expressed as follows:

(5) $$\begin{array}{c} ϒ=\left(Spatial\left(F_{S}\right)\times F_{S}\right)\times F_{E}\text{.} \end{array}$$

The final fusion feature *T* can be expressed as follows:

(6) $$\begin{array}{c} T=conv_{3\times 3}\left(P+ϒ\right)\text{.} \end{array}$$

### 3.3. Decoder and Features Aggregation Module (FAM)

**Figure 4:**
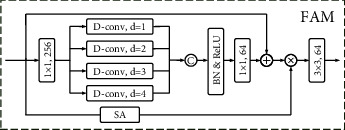
FAM: features aggregation module. SA: spatial attention. D-conv denote deep separable convolution, and d is dilation rate.

As shown in Figure 4, FAM utilizes multiscale learning (dilated convolution with different dilation rates) to expand the receptive field, enhance the boundary details of salient objects, and promote the fusion of salient features and edge features. The input feature is denoted as *χ*. We expand the number of *χ* channels to *M* times by 1 × 1 convolution. The depth separable convolution with different expansion rates is used for multiscale learning. This process can be expressed as follows:

(7) $$\begin{array}{c} \chi_{1}=conv_{1\times 1}\left(\chi \right)\text{,}\\ \chi_{2}^{d_{1}}=conv_{3\times 3}^{d_{1}}\left(\chi_{1}\right)\text{,}\\ \chi_{2}^{d_{2}}=conv_{3\times 3}^{d_{2}}\left(\chi_{1}\right)\text{,}\\ \chi_{2}^{d_{3}}=conv_{3\times 3}^{d_{3}}\left(\chi_{1}\right)\text{,}\\ \chi_{2}^{d_{4}}=conv_{3\times 3}^{d_{4}}\left(\chi_{1}\right)\text{,}\\ \chi_{2}=ReLU\left(BN\left(concatenation\left(\chi_{2}^{d_{1}},\chi_{2}^{d_{2}},\chi_{2}^{d_{3}},\chi_{2}^{d_{4}}\right)\right)\right)\text{,} \end{array}$$

where *d*_1_, *d*_2_, *d*_3_, *d*_4_ are dilation rates, taken as 1, 2, 3, 4 here. BN is the abbreviation of batch normalization. Here we set up a residual connection, which can be expressed as follows:

(8) $$\begin{array}{c} \chi_{3}=conv_{1\times 1}\left(\chi_{2}\right)+\chi \text{.} \end{array}$$

Then, we apply a spatial attention to *χ* and obtain the attention vector *χ*′. The feature of the final output can be expressed as follows:

(9) $$\begin{array}{c} \chi^{\prime }=Spatial\left(\chi \right)\times \chi \text{,}\\ out=conv\left(\chi_{3}\times \chi^{\prime }\right)\text{.} \end{array}$$

We obtain the final saliency prediction map from top to bottom through FAM. We added depth supervision (purple arrow in Figure 2) after the four FAM modules to refine the saliency map by learning the error between the saliency map and the ground truth.

### 3.4. Loss Function

The total loss function of our network consists of edge loss*ℒ*_*e*_and saliency loss*ℒ*_*S*_. Assume that *G*, *G*_*e*_ represents supervision from saliency ground-truth and edge ground-truth, and *P*_*k*_^*e*^and*P*_*k*_^*S*^ represent the edge prediction map and the saliency prediction map. The total loss function can be expressed as follows:

(10) $$\begin{array}{c} L_{total}=\overset{4}{\underset{k=1}{\sum }}L_{e}\left(P_{k}^{e},G_{e}\right)+\overset{9}{\underset{k=1}{\sum }}L_{S}\left(P_{k}^{S},G\right)\text{,} \end{array}$$

*ℒ*_*e*_and*ℒ*_*S*_uses the widely used cross-entropy loss function:

(11) $$\begin{array}{c} L\left(P,G\right)=-\underset{i}{\sum }\left[G_{i}\log\left(P_{i}\right)+\left(1-G_{i}\right)\log\;\left(1-P_{i}\right)\right]\text{,} \end{array}$$

where *i* represents the pixel index, *P* ∈ {*P*_*k*_^*e*^, *P*_*k*_^*S*^}.

## 4. Experiment

### 4.1. Datasets

We train our network on the subset DUTS-TR in the dataset DUTS [39]. We have evaluated the proposed network on six standard benchmark datasets: DUT-OMRON [40], DUTS [39], ECSSD [41], PASCAL-S [42], HKU-IS [43], and SOD [44, 45]. DUT-OMRON contains 5168 high-quality images. There are one or more salient objects with a relatively complex background structure. DUTS is so far the largest salient object detection dataset available. DUTS contains two subsets: training subset DUTS-TR and test subset DUTS-TE. DUTS-TR has 10553 images designed for training, and DUTS-TE has 5019 images designed for testing. ECSSD contains 1000 meaningful and complex semantic images with various complex scenes. PASCAL-S has 850 images with chaotic background and complex foreground. HKU-IS has 4447 images with high-quality annotations. Most images in the dataset have multiple connected or disconnected salient objects. SOD contains 300 high-quality images with a complex background. It was originally designed for image segmentation [44]. Pixel-level annotations of salient objects are generated in [45] and used for object detection. Although the SOD dataset has fewer images, it is currently one of the most challenging object detection datasets, since most of the images contain multiple salient objects, and some salient objects overlap with the boundary or have low contrast.

### 4.2. Experimental Details

We train our network on the DUTS-TR dataset. We use vggnet16 as the backbone network. All weights of the newly added convolutional layer are randomly initialized with truncated normal (*σ* = 0.01), and the deviation is initialized to 0. The hyperparameters of our network model are set as follows: learning rate = 2e-5, weight decay = 0.0005, momentum = 0.9, batch-size = 8. Backpropagation is processed in a group of 50 images. We do not use the validation dataset during the training process. The model is trained for 30 epochs, and the learning rate after 15 epochs is divided by 10. We implement our network model based on the publicly available Pytorch framework. We use a GTX 2080ti GPU (12 GB RAM) to train and test our model.

### 4.3. Evaluation Metrics

We use some widely used standard metrics, including F-measure, Mean Absolute Error (MAE) [7] and S-measure [46], and the PR curve, to evaluate our model and other advanced models. The PR curve is a standard method for evaluating the probability map of saliency prediction. It is actually a curve obtained by two variables, Precision (precision rate) and Recall (recall rate), where recall is the abscissa and precision the ordinate.

F-measure is an overall performance measurement, computed from the weighted harmonic mean of precision and recall. It is expressed as follows:

(12) $$\begin{array}{c} F_{\beta }=\frac{\left(1+\beta^{2}\right)\times Precision\times Recall}{\beta^{2}\times Precision\times Recall}\text{,} \end{array}$$

*β* ^2^ is set to 0.3 to weight precision more than recall.

The MAE value represents the average absolute pixel difference between the saliency map (represented by *S*) and the ground truth map (represented by *G*). It is expressed as follows:

(13) $$\begin{array}{c} MAE=\frac{1}{W\times H}\overset{W}{\underset{x=1}{\sum }}\overset{H}{\underset{y=1}{\sum }}\left|S\left(x,y\right)-G\left(x,y\right)\right|\text{,} \end{array}$$

where *W* and *H* represent the width and height of the saliency map, respectively.

S-measure focuses on evaluating the structural information of saliency maps. S-measure is closer to the human visual system than F-measure. The S-measure could be computed as follows:

(14) $$\begin{array}{c} S=\gamma S_{0}+\left(1-\gamma \right)S_{\gamma }\text{,} \end{array}$$

where *S*_0_ and *S*_*γ*_ denote the region-aware and object-aware structural similarity. *γ* is set as 0.5 by default.

### 4.4. Ablation Experiment and Analysis

In this section, we use DUTS-TR as the training set to verify the effectiveness of the key components in the proposed network. We also discuss the effects of different components in the proposed network on different datasets.

The baseline model is an encoder-decoder structure. It can integrate multi-scale features. We adopt saliency supervision and the cross-entropy loss function in this model. From Table 1, the U-shaped network built with vgg16 still has excellent performance.

**Figure 5:**
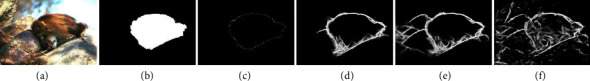
Examples of a visual edge map of ablation experiment. (a) RGB (b) GT (c) Edge GT (d) Base + U-E-G (e) Base + U-E (f) Base + E.

The Base + E model adds edge supervision to the side path2 of the Baseline model. The saliency prediction map is obtained by fusing salient edge features and salient features. As shown in Figure 5(f), there is a lot of redundant information in the edge features of the picture. From Table 1, after incorporating edge information into the Baseline model, the evaluation metrics are improved.

The Base + U-E model uses a U-shaped structure to extract edge features and fuses salient features and edge features by adding elements. Figure 5(e) is the obtained feature prediction map at the largest resolution among the four different resolutions. Compared with Figures 5(f), 5(e) has clearer object boundaries and less redundant information. Although the edge map obtained by Base + U-E model is finer than that obtained by Base + E model, from the evaluation metrics in Table 1, their SOD tasks do not differ significantly. Therefore, the network has to be further optimized.

Base + U-E-G model adds a GGM module to the Base + U-E model. Although the saliency map obtained by GGM has blurred boundaries (see Figure 2 coarse prediction map), its spatial location information is the most abundant. The predicted coarse saliency map serves as guidance to enhance the saliency information of the side output feature. By fusing the top-level semantic feature, the edge feature extracted by the U-shaped network is even finer, as shown in Figure 5(d). The evaluation metrics are also significantly improved.

Our final model adds FFM and FAM modules to the Base + U-E-G model. From the data in Table 1, through the optimization of FFM and FAM, our model has the best performance. This verifies that the proposed FFM and FAM modules can more effectively promote the fusion of edge features and salient features to improve performance.

As shown in Table 2, experiments are conducted using different feature fusion methods on SOD, HKU-IS, and PASCAL-S datasets. Method (a) uses elementwise addition instead of FFM to fuse salient features and edge features. Method (b) uses elementwise multiplication instead of FFM. Method (c) concatenates the two feature maps and performs a convolution operation to fuse the features. Method (d) utilizes a spatial attention module after using element addition for feature fusion. Compared with method (a), method (d) has improved performance after increasing spatial attention. In our model (e), FFM is a combination of elementwise addition and multiplication, and convolution and spatial attention. Comprehensively comparing the indicators of these datasets, our FFM module performs best.

### 4.5. Comparison with State-of-the-Arts

In this section, we compare our proposed MRBENet with 16 state-of-the-art methods, including AFNet [47], BMPM [20], BASNet [23], EGNet [24], PoolNet [26], Picanet [21], RAS [48], CPD [38], ASNet [49], Gatenet [50], ICON [51], CII [52], Auto-MSFNet [53], MINet [54], U2Net [55], and DNA [56]. For a fair comparison, we either use the saliency map provided by the author or run their source codes to get the saliency map.

**Figure 6:**
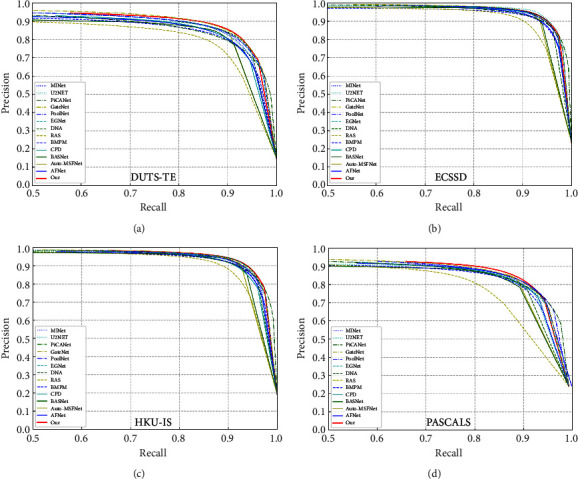
PR curve.

Quantitative evaluation. We evaluate our model MRBENet with other advanced models on six datasets. As shown in Table 3, we can see the MAE, Max F-measure, and S-measure values of different methods in different datasets. We draw PR curves of the different methods in Figure 6. Combining the graphs and tables, it can be seen that our method outperforms most methods. Our vgg16-based model has better performance than some Resnet-based models such as CPD and BASNet. After replacing the backbone network with resnet50, the performance of the model is improved.

**Figure 7:**
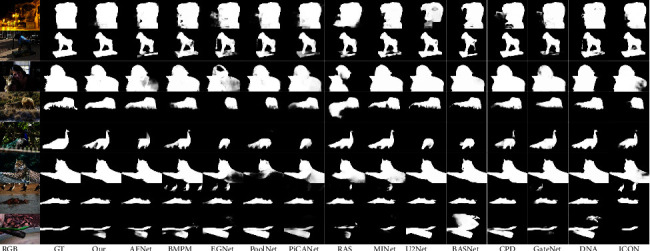
Qualitative comparison of our method with nine other methods.

Visual comparison. In Figure 7, we show the visualization results of different methods. It can be seen that our method performs well on images with low contrast (rows 1 to 3), complex background (rows 4 to 6), blurred borders (rows 1 to 5), and multiple objects (rows 7 to 8). Our method makes full use of high-level semantic information and edge information, and can still recognize salient objects in complex scenes.

## 5. Conclusion

In this paper, we propose an MRBENet network that enhances the fineness of salient objects through the multiscale fusion of salient edge features. The GGM incorporated into the backbone network can extract high-level semantic features, which can help locate object boundaries accurately for shallow features. The FFM fuses edge features and salient object features to enhance the edge of salient objects. Our model performs well against the state-of-the-art methods on six datasets. The experimental results show that the model can improve the salient object localization and edge fineness although the images have complex backgrounds and low contrast. In the future, we will continue to explore how to use edge information to improve saliency detection performance.

## Figures and Tables

**Table 1:** Ablation experiment was performed on SOD, HKU-IS, and DUTS-TE datasets.

Model	SOD			HKU-IS			DUTS-TE		
	MAE↓	MaxF↑	S↑	MAE↓	MaxF↑	S↑	MAE↓	MaxF↑	S↑
(a) Baseline	0.118	0.845	0.784	0.042	0.924	0.905	0.054	0.853	0.860
(b) Base + E	0.111	0.859	0.785	0.039	0.924	0.905	0.048	0.870	0.871
(c) Base + U-E	0.110	0.858	0.781	0.038	0.923	0.904	0.045	0.871	0.872
(d) Base + U-E-G	0.106	0.862	0.792	0.034	0.929	0.911	0.041	0.880	0.882
(e) Our model	0.103	0.866	0.791	0.032	0.933	0.913	0.040	0.878	0.881

**Table 2:** Ablation experiment for feature fusion.

Method	SOD			HKU-IS			PASCAL-S		
	MAE↓	MaxF↑	S↑	MAE↓	MaxF↑	S↑	MAE↓	MaxF↑	S↑
(a) ADD	0.0991	0.8672	0.7926	0.0307	0.9363	0.9186	0.0658	0.8717	0.8616
(b) Multiplication	0.1002	0.8679	0.7945	0.0309	0.9366	0.9188	0.0666	0.8739	0.8614
(c) Concat + conv	0.0997	0.8707	0.7943	0.0301	0.9365	0.9185	0.0663	0.8711	0.8605
(d) ADD + SA	0.0979	0.8670	0.7955	0.0307	0.9365	0.9186	0.0650	0.8761	0.8613
(e) Our model	0.0966	0.8695	0.8019	0.0299	0.9354	0.9181	0.0643	0.8764	0.8613

**Table 3:** Quantitative comparison of MaxF, MAE and S-measure over six widely used datasets. ↑ means bigger is better, ↓ means smaller is better. Red and blue represent the best and second best results, respectively. “-R” indicates that backbone network is Resnet.

	DUTS-TE			DUT-O			SOD			ECSSD			PASCAL-S			HKU-IS		
	MAE↓	MaxF↑	S↑	MAE↓	MaxF↑	S↑	MAE↓	MaxF↑	S↑	MAE↓	MaxF↑	S↑	MAE↓	MaxF↑	S↑	MAE↓	MaxF↑	S↑
AFNet(19)	0.045	0.857	0.866	0.057	0.787	0.823	0.108	0.851	0.779	0.042	0.935	0.914	0.070	0.863	0.849	0.036	0.923	0.905
BMPM(18)	0.048	0.847	0.861	0.063	0.765	0.807	0.106	0.852	0.790	0.045	0.929	0.911	0.074	0.850	0.844	0.039	0.921	0.906
EGNet(19)	0.043	0.871	0.877	0.056	0.798	0.832	0.110	0.859	0.787	0.041	0.943	0.919	0.077	0.859	0.847	0.034	0.930	0.911
PoolNet(19)	0.041	0.870	0.878	0.056	0.796	0.830	0.105	0.866	0.797	0.042	0.942	0.917	0.072	0.865	0.852	0.033	0.931	0.911
Picanet(18)	0.053	0.847	0.858	0.067	0.788	0.819	0.102	0.850	0.789	0.047	0.931	0.913	0.078	0.856	0.847	0.042	0.921	0.905
RAS(19)	0.059	0.827	0.839	0.062	0.778	0.813	0.123	0.847	0.767	0.056	0.921	0.893	0.101	0.829	0.799	0.045	0.913	0.887
MINet(20)	0.040	0.871	0.874	0.057	0.784	0.819	—	—	—	0.037	0.944	0.919	0.065	0.865	0.854	0.032	0.930	0.911
U2Net(20)	0.044	0.867	0.873	0.054	0.812	0.844	0.106	0.859	0.789	0.033	0.951	0.928	0.074	0.859	0.844	0.031	0.935	0.916
ASNet(20)	—	—	—	—	—	—	0.105	0.857	0.799	0.047	0.932	0.915	0.070	0.867	0.860	0.041	0.923	0.906
CPD-R(19)	0.043	0.861	0.869	0.056	0.790	0.823	0.110	0.857	0.771	0.037	0.939	0.918	0.071	0.860	0.848	0.034	0.925	0.905
BASNet-R(19)	0.047	0.855	0.865	0.057	0.796	0.833	0.112	0.849	0.772	0.037	0.943	0.916	0.076	0.854	0.838	0.032	0.928	0.908
Gatenet-R(20)	0.040	0.882	0.884	0.055	0.807	0.835	0.099	0.873	0.801	0.041	0.945	0.920	0.068	0.869	0.857	0.034	0.934	0.915
MSFNet(21)	0.039	0.866	0.870	0.056	0.790	0.821	—	—	—	0.036	0.937	0.913	0.067	0.858	0.852	0.029	0.936	0.905
CII-R(21)	0.042	0.878	0.874	0.058	0.824	0.828	—	—	—	0.039	0.941	0.916	0.068	0.868	0.851	0.032	0.933	0.912
ICON(22)	0.042	0.883	0.874	0.064	0.817	0.826	0.089	0.872	0.806	0.037	0.945	0.915	0.064	0.878	0.855	0.032	0.936	0.908
DNA-R(21)	0.040	0.869	0.872	0.056	0.796	0.824	0.110	0.855	0.783	0.036	0.944	0.919	0.074	0.855	0.837	0.029	0.934	0.913
MRBENet	0.040	0.878	0.881	0.054	0.806	0.835	0.103	0.866	0.791	0.037	0.945	0.922	0.067	0.872	0.857	0.032	0.933	0.913
MRBENet-R	0.037	0.882	0.887	0.052	0.806	0.837	0.097	0.870	0.802	0.036	0.945	0.923	0.064	0.876	0.861	0.030	0.935	0.918

## Data Availability

The data used to support the findings of this study are available from the corresponding author upon request.
